# Is there evidence to use kinematic/kinetic measures clinically in low back pain patients? A systematic review

**DOI:** 10.1016/j.clinbiomech.2018.04.006

**Published:** 2018-06

**Authors:** Enrica Papi, Anthony M.J. Bull, Alison H. McGregor

**Affiliations:** a Department of Surgery and Cancer, Imperial College London, London, UK; b Department of Bioengineering, Imperial College London, London, UK

**Keywords:** Low back pain, Movement, Functional assessment, Motion analysis, Objective measure

## Abstract

**Background:**

Currently, there is a widespread reliance on self-reported questionnaires to assess low back pain patients. However, it has been suggested that objective measures of low back pain patients' functional status should be used to aid clinical assessment. The aim of this study is to systematically review which kinematic /kinetic parameters have been used to assess low back pain patients against healthy controls and to propose clinical kinematic/kinetic measures.

**Methods:**

PubMed, Embase and Scopus databases were searched for relevant studies. Reference lists of selected studies and hand searches were performed. Studies had to compare people with and without non-specific low back pain while performing functional tasks and report body segment/joint kinematic and/or kinetic data. Two reviewers independently identified relevant papers.

**Findings:**

Sixty-two studies were included. Common biases identified were lack of assessor blinding and sample size calculation, use of samples of convenience, and poor experimental protocol standardization. Studies had small sample sizes. Range of motion maneuvers were the main task performed (33/62). Kinematic/kinetic data of different individual or combination of body segments/joints were reported among the studies, commonest was to assess the hip joint and lumbar segment motion (13/62). Only one study described full body movement. The most commonly reported outcome was range of motion. Statistically significant differences between controls and low back pain groups were reported for different outcomes among the studies. Moreover, when the same outcome was reported disagreements were noted.

**Interpretation:**

The literature to date offers limited and inconsistent evidence of kinematic/kinetic measures in low back pain patients that could be used clinically.

## Highlights

• Current low back pain assessment relies on subjective measures.
• Use of objective measures (kinematics/kinetics) is encouraged in low back pain assessment.
• This review highlights the heterogeneity of studies with low back pain and healthy subjects.
• There is poor evidence for kinematic/kinetic parameters as low back pain objective measures.
• Functional tasks, whole body and not only range of motion should be considered.

## 1 Introduction

Treatment for low back pain (LBP) aims to restore normal movement function and relieve pain. Measurements of movement function and measures of pain reduction, should, therefore, be the focus of LBP evaluation (Newman et al., 1996). This review is focused on measures of movement function. Movement analysis, allowing quantification of human movement, provides a means to objectify impairments from which clinical decisions can be made (Andriacchi and Alexander, 2000). However, clinical assessment of LBP relies predominately on self-reported questionnaires and scores, which depend on the patients' perception of their pain and functional capacity (Smeets et al., 2011). In many cases of LBP, the origin of pain cannot be identified, with diagnosis occurring in only 5–10% of cases (Krismer and van Tulder, 2007). This relates to the multifactorial and complex nature of LBP. Psychosocial factors, such as fear avoidance, dissatisfaction at work and pain beliefs as well as mechanical factors due to daily movement contribute to LBP development and occurrence (Clays et al., 2007). The interaction among these factors makes non-specific LBP difficult to classify and leaves clinicians facing significant challenges during its evaluation and management with consequences on patients' recovery. Imaging techniques, such as X-rays, computed tomography and magnetic resonance imaging, are employed in clinical practice but do not increase clinicians' ability to assess function and provide few if any indicators on how to manage non-specific LBP (Newman et al., 1996). Conversely, the ability to objectively assess the extent of movement impairments due to LBP has the potential to aid clinical assessment and, combined with psychosocial intervention, may provide important treatment targets.

The use of objective measures of LBP patients' movement function, alongside self-reported questionnaires, has been recently encouraged (Sanchez-Zuriaga et al., 2011; Smeets et al., 2011), yet definition of functional motion and what should be measured is lacking. Lumbar range of motion (RoM) is frequently used in the clinical diagnosis of LBP despite its known variability and its questionable ability to discriminate between controls and LBP patients (Laird et al., 2014; Lehman, 2004). Failure in differentiating these two groups on the basis of movement function is further aggravated by not considering the existence of sub-groups of LBP patients based on adopted movement strategies to accomplish a task. Moreover, it has been recently suggested that assessment should not be limited to the spine but should consider the spine in a whole-body context, including the lower limbs (McGregor and Hukins, 2009; Song et al., 2012). The lower limbs interfacing with externally applied forces may play an important role in spinal function during movement and standing as these are part of the body's kinematic chain. However, to date the full role of lower limb mechanics in the development and persistence of LBP is not known (McGregor and Hukins, 2009; Song et al., 2012). Since both the upper and lower body systems are active segments responsible for the achievement of everyday motor tasks none of them should be omitted in functional assessments.

For this paper, we focus on objective measures of LBP movement function that could empirically, by appropriate techniques, highlight significant differences between control and LBP populations thus providing a greater understanding of LBP biomechanical mechanisms to refine assessments and treatment options. This is to go beyond the subjectivity of self-reported questionnaires and observational clinical assessment. The aim of this systematic review was to evaluate the available literature in relation to kinematic and kinetic parameters that have been used to assess LBP patients' movement function compared to healthy controls and to identify possible objective measures of LBP, based on the parameters reported in published studies, which could be used clinically to aid LBP assessment and management. The research questions we sought to address were: i) Can kinematic/kinetic data differentiate between LBP patients and control subjects? ii) Which measurements and methods have been used to characterise patterns of motion that might be relevant to LBP? iii) Can such methods be translated to the clinical environment?

## 2 Methods

This systematic review was conducted in accordance with the PRISMA Statement (Moher et al., 2009).

### 2.1 Eligibility criteria

Studies were included in the review if they: 1) included adults over 18 years old, 2) were published in English, 3) considered patients presenting with non-specific LBP only, 4) included data from a healthy control group or healthy database, 5) used joint/body segment kinematic and/or kinetic data as an outcome measure, 6) considered active movements, 7) included appropriate statistical reporting, and 8) were peer-reviewed. Studies were excluded if they: 1) were a case-study design, 2) included subjects with specific LBP caused by pathological entities and attributable to a recognisable pathology (e.g., scoliosis, spinal stenosis, disc herniation, ankylosing spondylitis, cauda equina, tumour, osteoporosis, fracture), 3) reported only imaging or muscle data, and 4) described patients as having back pain with no specific reference made to LBP.

### 2.2 Data sources and search strategy

Electronic databases, PubMed, Embase and Scopus, were searched from the earliest records up until May 2016. The search strategy combined three conceptual groups of terms: LBP, Testing Procedure/Method, and Measurement/Outcome. Controlled vocabulary terms (e.g. Mesh terms) and key words were used. PubMed search strategy, from which other database searches were derived, is reported in Supplementary File 1. Citation tracking of selected studies and hand searches were also performed to identify additional relevant articles missed by the electronic searches. Searched articles were imported into EndNote ×7 software (Thomson, Reuters, Carlsbad, CA) for subsequent study selection.

### 2.3 Review process

Two independent reviewers (EP, AM) screened titles and abstracts to identify eligible studies. Full text articles were assessed for eligibility criteria by EP and AM independently. Disagreements were resolved by consensus discussions.

**Table 1:** Quality assessment summary.

Quality assessment domains		% of studies scoring yes
Study population bias		
1	Was the study population adequately described?	85%
2	Were both groups drawn from the same population?	16%
3	Were both groups comparable for age, sex, BMI/weight?	72%
4	Were the subjects asked to participate in the study representative of the entire population from which they were recruited?	0%
5	Was pain intensity and/or activity limitation described for LBP group?	72%
6	Was an attempt made to define back pain characteristics?	92%
7	Were the eligibility criteria specified?	89%

Measurement and outcome bias		
8	Did the method description enable accurate replication of the measurement procedures?	98%
9	Was the measurement equipment adequately described?	100%
10	Was a system for standardizing movement instructions reported?	42%
11	Were assessors trained in standardized measurement procedure?	8%
12	Did the same assessors test those with and without back pain?	11%
13	Were assessors blinded as to which group subjects were in?	2%
14	Was assessment procedure applied to those with and without back pain the same?	100%
15	Were the main outcomes to be measured and the related calculations (if applicable) clearly described?	97%
16	Were the main outcome measures used accurate (valid and reliable)?	97%

Data presentation bias		
17	Are the main findings of the study clearly described?	97%
18	Were the statistical tests appropriate?	98%
19	The results of between-group statistical comparisons were reported for at least one key outcome	95%
20	Have actual probability values been reported (e.g. 0.035 rather than <0.05) for the main outcomes except where the probability value is <0.001?	53%
21	Point estimates and measures of variability were provided for at least one key outcome for those with and without back pain	92%
22	Did the study have sufficient power to detect a clinically important effect where the probability value for a difference being due to chance is <5%?	10%
23	Was the reliability and/or validity of the outcomes commented upon?	56%

### 2.4 Data extraction and quality assessment

The following study details were extracted from each included study, using a customised data extraction form: study aims, design, sample size, participant demographics, task conducted, equipment used, body segments analysed, kinematic and kinetic variables evaluated, statistical analysis technique, statistically significant outcomes. As no standardized or validated quality checklists exist for this type of review, a customised quality assessment tool was constructed based on tools used in similar studies (Downs and Black, 1998; Laird et al., 2014) to determine sources of bias in the selected articles. The quality assessment tool used was divided into three domains: study population bias, measurement and outcome bias, and data presentation bias (Table 1). Population description, experimental methodology and reporting of the results could, thus, be evaluated. Ratcliffe et al.'s rating score was used to rate the quality of the reviewed paper: studies scored as high quality achieve a score >66.8%, medium quality 33.4–66.7%, and low quality <33.3%. (Ratcliffe et al., 2014). Assessment checklist questions and the correspondent decision rules are available in Supplementary File 2.

## 3 Results

### 3.1 Study selection

**Fig. 1:**
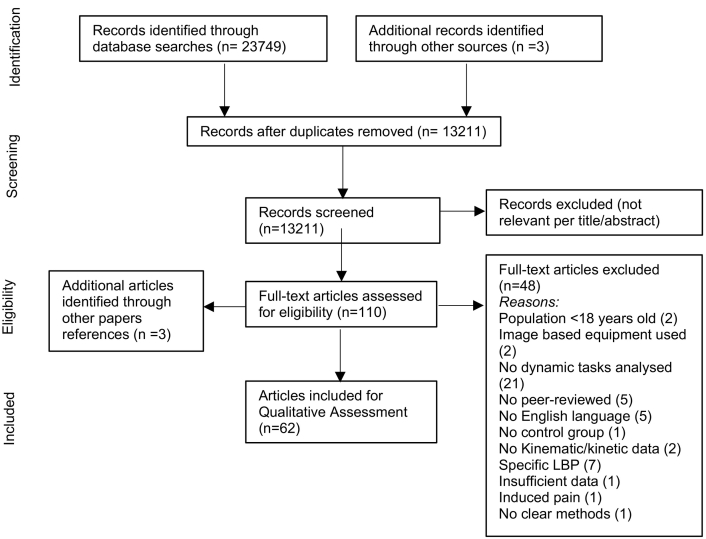
PRISMA flow diagram illustrating the review process (Moher et al., 2009).

The study selection process is shown in Fig. 1. The initial search yielded 13,211 articles, with duplicates removed, with 6 additional articles identified through citation tracking and hand searches. After screening titles and abstracts, 13,104 articles were excluded as they were deemed irrelevant to this review topic. Inclusion criteria were applied to the full-texts of 110 articles. Of these, 62 met the inclusion criteria; reasons for exclusion of the other 48 articles are shown in Fig. 1. A meta-analysis of the study results was not appropriate as this review did not examine clinical interventions and also because of the diverse methodological approaches adopted. A summary of included studies is available in Supplementary File 3.

### 3.2 Quality aspects of reviewed studies

Six studies had a quality score below 50% (Akinpelu and Adeyemi, 1989; Aluko et al., 2011; Cyteval et al., 1996; Lamoth et al., 2002; McGregor et al., 1997; Newman et al., 1996). The highest score recorded was 78% in 4 studies (Burnett et al., 2004; Jayaraman et al., 1994; Song et al., 2012; Sung et al., 2012). Based on Ratcliffe et al.'s (Ratcliffe et al., 2014) rating score, 27 articles [10, 19–43] were of high, and the remaining 35 articles (Akinpelu and Adeyemi, 1989; Al-Eisa et al., 2006a; Al-Eisa et al., 2006b; Aluko et al., 2011; Barrett et al., 1999; Boline et al., 1992; Crosbie et al., 2013a; Crosbie et al., 2013b; Cyteval et al., 1996; Fenety and Kumar, 1992; Freddolini et al., 2014a; Freddolini et al., 2014b; Gioftsos and Grieve, 1996; Henchoz et al., 2013; Jandre Reis and Macedo, 2015; Kim et al., 2013; Lamoth et al., 2002; Lamoth et al., 2006b; Lee et al., 2011b; McClure et al., 1997; McGregor et al., 1997; Morlock et al., 2000; Muller et al., 2015; Newman et al., 1996; Ng et al., 2002; Porter and Wilkinson, 1997; Shum et al., 2005a; Shum et al., 2005b; Shum et al., 2007b; Vaisy et al., 2015; van Wingerden et al., 2008; Vismara et al., 2010; Vogt et al., 2001; Vogt et al., 2003; Wong and Lee, 2004) were of medium quality. Table 1 shows a quality assessment summary of all included studies indicating potential sources of bias. Ten studies (Burnett et al., 2004; Dunk and Callaghan, 2010; Esola et al., 1996; Mellin, 1990; Mitchell et al., 2008; Morlock et al., 2000; Muller et al., 2015; Seay et al., 2011; Song et al., 2012; Sung et al., 2012; Van Hoof et al., 2012) used participants from the same population group; none identified the source population for participants, only 1 study (Newman et al., 1996) blinded the assessors to group status and 5 studies (Boline et al., 1992; Gombatto et al., 2015; Jayaraman et al., 1994; Park et al., 2012; Vismara et al., 2010) gave evidence of assessors' expertise. Seven studies (Barrett et al., 1999; Boline et al., 1992; Jayaraman et al., 1994; Mellin, 1990; Newcomer et al., 2000; Porter and Wilkinson, 1997; Vismara et al., 2010) described if the tests were conducted by the same assessor and 26 reported the use of standardized movement instructions (Al-Eisa et al., 2006a; Al-Eisa et al., 2006b; Barrett et al., 1999; Boline et al., 1992; Burnett et al., 2004; Crosbie et al., 2013a; Crosbie et al., 2013b; Dankaerts et al., 2009; Esola et al., 1996; Gioftsos and Grieve, 1996; Gombatto et al., 2015; Jayaraman et al., 1994; Kim et al., 2013; Kim et al., 2014; Kim and Yoo, 2015; Lamoth et al., 2002; Lamoth et al., 2006b; Lariviere et al., 2000; Larivière et al., 2002; Larivière et al., 2011; Mitchell et al., 2008; Park et al., 2012; Sanchez-Zuriaga et al., 2011; Song et al., 2012; Sung, 2013; Sung et al., 2012). Only 6 justified their sample size (Jayaraman et al., 1994; Larivière et al., 2011; Mitchell et al., 2008; Newcomer et al., 2000; Seay et al., 2011; Shum et al., 2007a).

### 3.3 Study characteristics

**Fig. 2:**
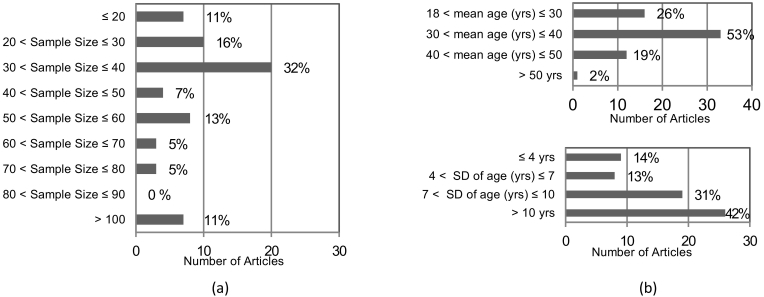
(a) Overall sample size, (b) participants mean age (top) and age variability expressed as standard deviation (SD) per number of selected articles. In brackets corresponding % of articles is shown.

Studies included in the review were published between 1989 and 2015, with the majority published in the last 15 years (49/62) (Al-Eisa et al., 2006a; Al-Eisa et al., 2006b; Aluko et al., 2011; Burnett et al., 2004; Crosbie et al., 2013a; Crosbie et al., 2013b; Dankaerts et al., 2009; Dunk and Callaghan, 2010; Freddolini et al., 2014a; Freddolini et al., 2014b; Gombatto et al., 2015; Henchoz et al., 2013; Jandre Reis and Macedo, 2015; Kim et al., 2013; Kim et al., 2014; Kim and Yoo, 2015; Lamoth et al., 2002; Lamoth et al., 2006a; Lamoth et al., 2006b; Larivière et al., 2011; Lee et al., 2011a; Lee et al., 2011b; Mitchell et al., 2008; Morlock et al., 2000; Muller et al., 2015; Ng et al., 2002; Park et al., 2012; Sanchez-Zuriaga et al., 2011; Seay et al., 2011; Shum et al., 2005a; Shum et al., 2005b; Shum et al., 2007a; Shum et al., 2007b; Song et al., 2012; Sung, 2013; Sung et al., 2012; Taylor et al., 2003; Taylor et al., 2004; Vaisy et al., 2015; van den Hoorn et al., 2012; Van Hoof et al., 2012; van Wingerden et al., 2008; Vismara et al., 2010; Vogt et al., 2001; Vogt et al., 2003; Wong and Lee, 2004). Most of the studies (34/62) (Boline et al., 1992; Crosbie et al., 2013a; Crosbie et al., 2013b; Cyteval et al., 1996; Dunk and Callaghan, 2010; Esola et al., 1996; Fenety and Kumar, 1992; Gioftsos and Grieve, 1996; Gombatto et al., 2015; Henchoz et al., 2013; Jayaraman et al., 1994; Kim et al., 2013; Kim et al., 2014; Kim and Yoo, 2015; Lamoth et al., 2006b; Lariviere et al., 2000; Larivière et al., 2002; Larivière et al., 2011; Lee et al., 2011a; McClure et al., 1997; Muller et al., 2015; Newcomer et al., 2000; Ng et al., 2002; Park et al., 2012; Porter and Wilkinson, 1997; Seay et al., 2011; Song et al., 2012; Sung, 2013; Sung et al., 2012; Taylor et al., 2004; Vaisy et al., 2015; van den Hoorn et al., 2012; Vismara et al., 2010; Vogt et al., 2003) had a small sample size with 11 to 25 participants in each group and 7 studies had a maximum of 10 participants per group (Fig. 2a) (Aluko et al., 2011; Burnett et al., 2004; Lee et al., 2011a; Lee et al., 2011b; Morlock et al., 2000; Taylor et al., 2003; Van Hoof et al., 2012).

Chronic LBP patients were recruited in 37 studies (Akinpelu and Adeyemi, 1989; Boline et al., 1992; Burnett et al., 2004; Crosbie et al., 2013a; Crosbie et al., 2013b; Cyteval et al., 1996; Dankaerts et al., 2009; Gioftsos and Grieve, 1996; Henchoz et al., 2013; Jandre Reis and Macedo, 2015; Jayaraman et al., 1994; Kim et al., 2014; Lamoth et al., 2002; Lamoth et al., 2006a; Lamoth et al., 2006b; Lariviere et al., 2000; Larivière et al., 2002; Larivière et al., 2011; Lee et al., 2011a; Lee et al., 2011b; McClure et al., 1997; Muller et al., 2015; Newcomer et al., 2000; Ng et al., 2002; Park et al., 2012; Porter and Wilkinson, 1997; Sanchez-Zuriaga et al., 2011; Seay et al., 2011; Sung, 2013; Vaisy et al., 2015; van den Hoorn et al., 2012; Van Hoof et al., 2012; van Wingerden et al., 2008; Vismara et al., 2010; Vogt et al., 2001; Vogt et al., 2003; Wong and Lee, 2004), 9 studies (Aluko et al., 2011; Freddolini et al., 2014a; Freddolini et al., 2014b; Shum et al., 2005a; Shum et al., 2005b; Shum et al., 2007a; Shum et al., 2007b; Taylor et al., 2003; Taylor et al., 2004) recruited acute LBP patients and 5 studies had a mix of chronic and acute LBP patients (Dunk and Callaghan, 2010; Esola et al., 1996; Gombatto et al., 2015; Song et al., 2012; Sung et al., 2012). In 11 articles the type of LBP (e.g. Chronic or Acute) was not conveyed (Al-Eisa et al., 2006a; Al-Eisa et al., 2006b; Barrett et al., 1999; Fenety and Kumar, 1992; Kim et al., 2014; Kim and Yoo, 2015; McGregor et al., 1997; Mellin, 1990; Mitchell et al., 2008; Morlock et al., 2000; Newman et al., 1996). Moreover, LBP duration was often lacking and large durations in symptoms were reported (ranging from 3 months to 5 years within one study). The level of pain/disability, if described (43/62), was low to moderate (Aluko et al., 2011; Burnett et al., 2004; Crosbie et al., 2013a; Crosbie et al., 2013b; Dankaerts et al., 2009; Dunk and Callaghan, 2010; Esola et al., 1996; Freddolini et al., 2014a; Freddolini et al., 2014b; Gioftsos and Grieve, 1996; Gombatto et al., 2015; Henchoz et al., 2013; Kim et al., 2014; Kim and Yoo, 2015; Lamoth et al., 2006a; Lee et al., 2011b; McClure et al., 1997; Mitchell et al., 2008; Muller et al., 2015; Newcomer et al., 2000; Ng et al., 2002; Park et al., 2012; Sanchez-Zuriaga et al., 2011; Seay et al., 2011; Shum et al., 2005a; Shum et al., 2005b; Shum et al., 2007a; Shum et al., 2007b; Song et al., 2012; Sung, 2013; Sung et al., 2012; Taylor et al., 2003; Taylor et al., 2004; Vaisy et al., 2015; van den Hoorn et al., 2012; Van Hoof et al., 2012; van Wingerden et al., 2008; Vogt et al., 2001; Vogt et al., 2003; Wong and Lee, 2004).

Most studies demonstrated an age bias with most participants recruited being in their thirties (Fig. 2b). It is however worth noticing the age variability within groups, in 45 studies age standard deviation values were above 7 years (Akinpelu and Adeyemi, 1989; Al-Eisa et al., 2006a; Al-Eisa et al., 2006b; Aluko et al., 2011; Barrett et al., 1999; Crosbie et al., 2013a; Crosbie et al., 2013b; Cyteval et al., 1996; Dankaerts et al., 2009; Esola et al., 1996; Freddolini et al., 2014a; Freddolini et al., 2014b; Gioftsos and Grieve, 1996; Gombatto et al., 2015; Henchoz et al., 2013; Lamoth et al., 2002; Lamoth et al., 2006a; Lamoth et al., 2006b; Larivière et al., 2011; Lee et al., 2011a; Lee et al., 2011b; McClure et al., 1997; McGregor et al., 1997; Newcomer et al., 2000; Newman et al., 1996; Sanchez-Zuriaga et al., 2011; Seay et al., 2011; Shum et al., 2005a; Shum et al., 2005b; Shum et al., 2007a; Shum et al., 2007b; Song et al., 2012; Sung, 2013; Sung et al., 2012; Taylor et al., 2003; Taylor et al., 2004; Vaisy et al., 2015; van den Hoorn et al., 2012; Van Hoof et al., 2012; van Wingerden et al., 2008; Vismara et al., 2010; Wong and Lee, 2004).

**Fig. 3:**
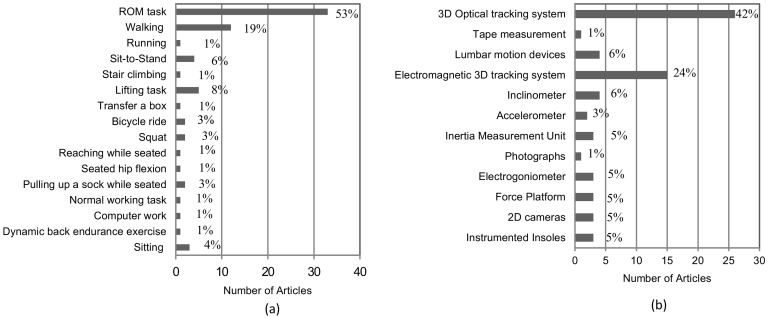
(a) Tasks evaluated and (b) equipment used per number of selected articles. In brackets corresponding % of articles is shown.

RoM maneuvers were the main tasks performed during the assessments (33/62) (Akinpelu and Adeyemi, 1989; Al-Eisa et al., 2006a; Al-Eisa et al., 2006b; Aluko et al., 2011; Barrett et al., 1999; Boline et al., 1992; Crosbie et al., 2013a; Cyteval et al., 1996; Dankaerts et al., 2009; Dunk and Callaghan, 2010; Esola et al., 1996; Fenety and Kumar, 1992; Henchoz et al., 2013; Jandre Reis and Macedo, 2015; Jayaraman et al., 1994; Kim et al., 2013; Kim and Yoo, 2015; Lariviere et al., 2000; Lee et al., 2011b; McClure et al., 1997; McGregor et al., 1997; Mellin, 1990; Mitchell et al., 2008; Newcomer et al., 2000; Ng et al., 2002; Park et al., 2012; Porter and Wilkinson, 1997; Song et al., 2012; Sung et al., 2012; Vaisy et al., 2015; van Wingerden et al., 2008; Vismara et al., 2010; Wong and Lee, 2004). Functional activities evaluated varied across studies; walking was the only task reported in >10 studies (Fig. 3a).

Three-dimensional (3D) tracking systems, either optical or electromagnetic, were the commonest tools used to assess movement (41/62, Fig. 3b) (Al-Eisa et al., 2006a; Al-Eisa et al., 2006b; Barrett et al., 1999; Burnett et al., 2004; Crosbie et al., 2013a; Crosbie et al., 2013b; Cyteval et al., 1996; Dankaerts et al., 2009; Esola et al., 1996; Freddolini et al., 2014a; Freddolini et al., 2014b; Gombatto et al., 2015; Henchoz et al., 2013; Jayaraman et al., 1994; Kim et al., 2013; Kim et al., 2014; Kim and Yoo, 2015; Lamoth et al., 2002; Lamoth et al., 2006a; Lamoth et al., 2006b; McClure et al., 1997; Mitchell et al., 2008; Muller et al., 2015; Newcomer et al., 2000; Newman et al., 1996; Park et al., 2012; Porter and Wilkinson, 1997; Sanchez-Zuriaga et al., 2011; Seay et al., 2011; Shum et al., 2005a; Shum et al., 2005b; Shum et al., 2007a; Shum et al., 2007b; Song et al., 2012; Sung, 2013; Sung et al., 2012; Taylor et al., 2003; Taylor et al., 2004; van den Hoorn et al., 2012; Vismara et al., 2010; Wong and Lee, 2004). More portable assessment devices that could be used outside of a laboratory comprised accelerometers, inertia measurement units, electrogoniometers and instrumented insoles (10/62) (Dunk and Callaghan, 2010; Gioftsos and Grieve, 1996; Larivière et al., 2011; Lee et al., 2011a; Lee et al., 2011b; Morlock et al., 2000; Taylor et al., 2003; Taylor et al., 2004; Van Hoof et al., 2012; Vogt et al., 2003). Some of the studies used more than one device to assess motion (e.g. motion capture with force plates).

**Fig. 4:**
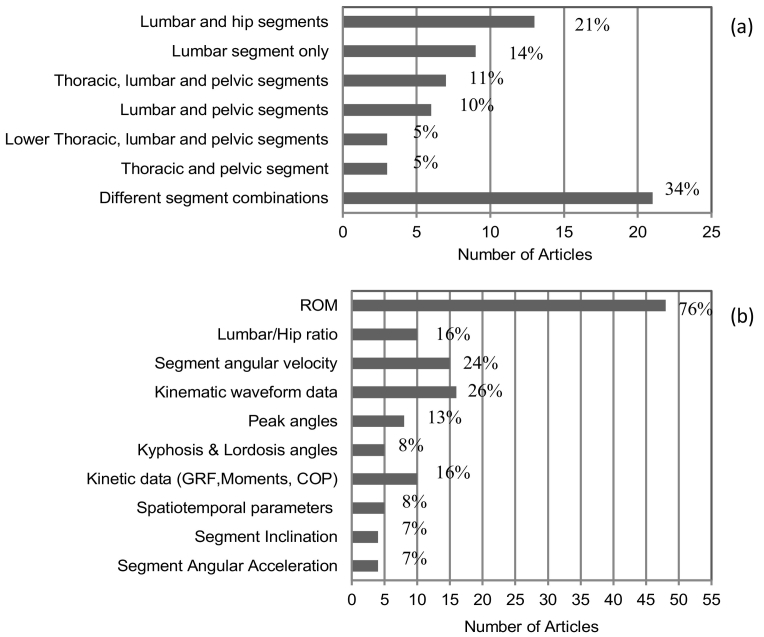
(a) Body segment/joint analysed and (b) outcome measures reported per number of selected articles. In brackets corresponding % of articles is show.

Diverse body segments/joints and combinations of body segments/joints were monitored (Fig. 4a). The combination of measuring lumbar segment motion, as the motion between the lumbar and pelvis segments and, hip joint motion, as the motion between the pelvis and thigh segments, was the commonest combination observed in 13 studies (Esola et al., 1996; Freddolini et al., 2014a; Freddolini et al., 2014b; Henchoz et al., 2013; Kim et al., 2013; McClure et al., 1997; Porter and Wilkinson, 1997; Shum et al., 2005a; Shum et al., 2005b; Shum et al., 2007a; Shum et al., 2007b; Sung, 2013; Wong and Lee, 2004); 9 studies (Akinpelu and Adeyemi, 1989; Aluko et al., 2011; Boline et al., 1992; Dunk and Callaghan, 2010; Fenety and Kumar, 1992; Kim and Yoo, 2015; McGregor et al., 1997; Newman et al., 1996; Ng et al., 2002) focused on solely the lumbar spine and the remainder did varying combinations of body regions. Only 1 study (Jayaraman et al., 1994) looked at full body movement and only 6 studies considered at least one of the lower limb joints other than the hip (Gioftsos and Grieve, 1996; Morlock et al., 2000; Muller et al., 2015; Newcomer et al., 2000; Sanchez-Zuriaga et al., 2011; Song et al., 2012). Only 5 studies considered the lumbar spine as a multi-segment model reporting upper and lower lumbar segments' motion as the relative movement between the upper and lower lumbar segments and lower lumbar and pelvis segments respectively (Burnett et al., 2004; Dankaerts et al., 2009; Gombatto et al., 2015; Jayaraman et al., 1994; Mitchell et al., 2008). None of the studies considered each vertebrae of the spine as a separate segment.

Regarding the outcomes used (Fig. 4b), the majority of studies (49/62) reported RoM values over the task performed (Akinpelu and Adeyemi, 1989; Al-Eisa et al., 2006a; Al-Eisa et al., 2006b; Barrett et al., 1999; Boline et al., 1992; Crosbie et al., 2013a; Crosbie et al., 2013b; Cyteval et al., 1996; Dankaerts et al., 2009; Dunk and Callaghan, 2010; Esola et al., 1996; Fenety and Kumar, 1992; Freddolini et al., 2014a; Freddolini et al., 2014b; Gioftsos and Grieve, 1996; Jandre Reis and Macedo, 2015; Kim et al., 2014; Kim and Yoo, 2015; Lamoth et al., 2002; Lamoth et al., 2006a; Lee et al., 2011a; Lee et al., 2011b; McClure et al., 1997; McGregor et al., 1997; Mellin, 1990; Mitchell et al., 2008; Muller et al., 2015; Newcomer et al., 2000; Ng et al., 2002; Park et al., 2012; Porter and Wilkinson, 1997; Sanchez-Zuriaga et al., 2011; Seay et al., 2011; Shum et al., 2005a; Shum et al., 2005b; Shum et al., 2007a; Song et al., 2012; Sung, 2013; Sung et al., 2012; Taylor et al., 2003; Taylor et al., 2004; Vaisy et al., 2015; Van Hoof et al., 2012; van Wingerden et al., 2008; Vismara et al., 2010; Vogt et al., 2001; Vogt et al., 2003; Wong and Lee, 2004); the joint angular time varying waveforms were shown in 16 studies (Al-Eisa et al., 2006a; Al-Eisa et al., 2006b; Crosbie et al., 2013b; Jayaraman et al., 1994; Kim et al., 2013; Lamoth et al., 2002; Lamoth et al., 2006a; Lee et al., 2011a; Lee et al., 2011b; McGregor et al., 1997; Muller et al., 2015; Park et al., 2012; Seay et al., 2011; Vaisy et al., 2015; van den Hoorn et al., 2012; Vogt et al., 2001). Kinetic data were reported in 7 studies (Freddolini et al., 2014b; Gioftsos and Grieve, 1996; Jayaraman et al., 1994; Larivière et al., 2002; Muller et al., 2015; Sanchez-Zuriaga et al., 2011; Shum et al., 2007b). It was common practice to describe movement in one anatomical plane, usually the sagittal plane (21/62) (Barrett et al., 1999; Cyteval et al., 1996; Dankaerts et al., 2009; Dunk and Callaghan, 2010; Esola et al., 1996; Fenety and Kumar, 1992; Freddolini et al., 2014a; Freddolini et al., 2014b; Gioftsos and Grieve, 1996; Henchoz et al., 2013; Jandre Reis and Macedo, 2015; Kim and Yoo, 2015; Larivière et al., 2011; McClure et al., 1997; Mitchell et al., 2008; Newman et al., 1996; Porter and Wilkinson, 1997; Shum et al., 2005a; Van Hoof et al., 2012; van Wingerden et al., 2008; Vogt et al., 2003).

### 3.4 Significant outcomes between control and LBP groups

Significant differences between controls and LBP groups were tabulated based on the type of outcomes and body segments/joints to which they referred. Wide diversity and poor consistency among the results were found, precluding a simple summary. More detailed descriptions of each study findings can be found in Supplementary File 3. Results of studies that do not report statistical differences between control and LBP groups can be found in Table 2 (Burnett et al., 2004; Cyteval et al., 1996; Esola et al., 1996; Freddolini et al., 2014b; Lamoth et al., 2002; Lamoth et al., 2006a; Lamoth et al., 2006b; Lariviere et al., 2000; Larivière et al., 2002; Morlock et al., 2000; Newcomer et al., 2000; Ng et al., 2002; Taylor et al., 2003; Taylor et al., 2004; Vogt et al., 2003). Differences identified in lumbar segment angles and lumbar/hip ratio are shown in Table 3. The lumbar/hip ratio was used to describe the contribution of both the lumbar segment and hip joint to the movement performed. Pelvis segment and shoulder, hip, knee angular results are reported in Table 4. Table 5 shows significant data for angles of the trunk, and thoracic spine.

Differences in kinetic data were reported in only a few studies: Shum et al. reported a reduced lumbar and hip extension moment, increased hip adduction and internal rotation moments and increased lumbar axial rotation moment(Vaisy et al., 2015); the latter finding was also reported by Lariviere et al. (Larivière et al., 2002). Two studies reported a decrease in the Ground Reaction Forces (GRF) (Muller et al., 2015; Sanchez-Zuriaga et al., 2011). Jayaraman et al. reported an increased medio/lateral GRF moment, a reduced antero/posterior GRF moment and an altered centre of pressure position that was more posteriorly displaced and closer to the body in LBP participants (Jayaraman et al., 1994). Angular speed and acceleration results for body segments/joints analysed are shown in Table 6. Some authors also developed classification models to discriminate between control and LBP groups using kinematic data only (Lehman, 2004; Newman et al., 1996; Sanchez-Zuriaga et al., 2011), combinations of kinematic and kinetic data (Gioftsos and Grieve, 1996) and kinematic with electromyography data (Dankaerts et al., 2009).

**Table 2:** Findings of reviewed articles that did not report statistically significant differences between LBP and control group (corresponding reference number given).

Article	Findings
16	*Dorsal flexion angle*: in control group there was a significant difference between the average angle at 0° and 90° flexion. No differences in LBP during motion indicating dorsal rigidity. Average value across groups 25°. *Lumbar flexion angle*: significantly different between women (27°) and men (21°) but no between groups. Average value across groups between 30° and 35°.
17	Mean transverse plane trunk ROM was 12 ± 4°for control group and 10 ± 4° for LBP group. Differences in movement coordination observed: relative Fourier phase (RFP) between pelvis and thorax increased with walking velocity and was higher in control group. LBP tended to preserve in-phase coordination at all velocities. Weighted coherence was smaller in LBP than control at low velocities indicating stronger coupling between pelvis and thorax.
20	There was a trend towards increased spinal flexion in the lower thoracic region at the start (control: 2.7 ± 5.9° vs LBP: 10.8 ± 10.9°) and finish (control: 3.8 ± 5.7° vs LBP: 11 ± 12.2°) of the ride and increased range of axial rotation in the lower lumbar spine for LBP group at the start of the ride (control: 2.2 ± 0.9° vs LBP:3.4 ± 1.8°). LBP displayed also larger lower lumbar flexion (control: 24.9 ± 20.2° vs LBP: 38.6 ± 19.9°).
28	LBP group showed higher ROM at the thoracic, lumbar and pelvis segments than controls in the transverse plane, whereas lower RoM in the frontal plane across all tests speeds (from 1.4 to 7 km/h). Maximum difference between groups was 3.1°. Principal Component Analysis showed that health status had no effect on the global kinematic walking pattern but indicated differences in the relative timing between the segment rotations. LBP tended to move the lumbar and pelvic segments more synchronously and rigidly in the same direction. Intersegmental coordination was higher in the transverse plane than in the frontal plane.
31	Pelvis, lumbar, and thoracic spine segments significantly changed their ROM across defined movement intervals but not in the same manner. The ROM of the lumbar spine monotonically decreased (11° change) across the intervals which was compensated by a gradual increase at the hip (2° change) and thoracic spine (7° change). A significant group x interval interaction was observed for the ROM of the lumbar spine, showing a faster ROM decrease across the four intervals in the control group (from 22° to 8°) than in LBP group (from 23° to 14°).
38	There was a trend of increasing pelvic obliquity to walk faster in LBP group(3.6 ± 2.7°) greater than when the pain resolved (6 weeks after initial test) (1.8 ± 1.3°) and in control group (2.7 ± 1.4°). The same was observed for lumbar lateral flexion: LBP 4 ± 2.8°; pain resolved 1.7 ± 1.9°, control 2.2 ± 1.6°. No kinematics differences but different strategies to achieve fast walking between groups.
39	Acute LBP group showed a trend towards larger 3D ROM at the pelvis and lumbar segment (both relative to the pelvis and global reference frame) when compared to controls walking at a matched speed and when the pain was resolved (6 weeks after initial test). Average difference between LBP and control 0.66 ± 0.5°.
43	Repositioning Error (RE) not significantly different between groups with mean values showing a trend to be higher in LBP for lumbar flexion, extension, right and left lateral bending, lower in right rotation and knee extension, and the same in left rotation. RE demonstrated differences due to direction of movement (*p* < 0.001)
52	Both lumbar RoM and moment tended to be higher in LBP with difference on average of 0.26 ± 0.3° and 5.26 ± 3.6 Nm.
57	Relative phases between thoracic and pelvic segment and lumbar and pelvic segment in the transverse plane decreased and increased respectively significantly with velocity; the velocity effect was less pronounced in LBP and was not observed in the frontal plane. Principal Component Analysis indicated that LBP presents with a reduced ability to adapt trunk-pelvis coordination after velocity perturbations and tend to move lumbar and pelvis segment as one rigid unit.
60	There was a trend in LBP group to greater mean joint moments at L5/S1 in the 3 anatomical planes, average difference of 3.03 ± 3.4 Nm. Mean bone -to-bone contact forces at L5/S1 was the same in both groups 2.4BW, the maximum value was 9.7 ± 1 BW and 10.5 ± 0.9 BW in LBP and control, respectively. For further analysis measures between the groups were averaged as no statistical differences found. (BW: Body Weight)
62	Only flexion lumbar ROM and lumbar lordosis were higher in the LBP compared to the control group, all other measurements (lumbar extension, lateral flexion and axial rotation RoM) were higher in the controls, differences between groups were of 1 to 2°.
70	Consistent kinematic patterns at the pelvis and thoracolumbar segment observed between LBP and control (*r* = 0.78 and 0.98 respectively). LBP group displayed significantly greater movement variability in all 3 anatomical planes when compared to control. ROM differences were on average −0.1 ± 0.5° at the sacrum and 0.5 ± 0.7° at the thoracolumbar segment.

RoM:Range of Motion.

**Table 3:** Significant lumbar segment angles and lumbar/hip ratio values reported in the selected studies (corresponding reference number given).

Lumbar segment					
	Sagittal plane				
		Total RoM			
		Decreased	Fenety and Kumar, 1992, Freddolini et al., 2014a, Lee et al., 2011a, Sung, 2013, Vaisy et al., 2015		
			Extension RoM		
			Decreased	Fenety and Kumar, 1992, Lee et al., 2011b, Mellin, 1990 (male only), Shum et al., 2005a	
			Different (values not shown)	Mitchell et al., 2008	
			Flexion RoM		
			Decreased	Akinpelu and Adeyemi, 1989, Porter and Wilkinson, 1997, Sanchez-Zuriaga et al., 2011, Shum et al., 2005a, Shum et al., 2005b, Shum et al., 2007a, Vismara et al., 2010 (at max flex), Wong and Lee, 2004	
				Lower lumbar segment	
				Total RoM	
				Decreased	Dankaerts et al., 2009 (active extension pattern LBP)
				Increased	Van Hoof et al., 2012

	Coronal plane (lateral flexion)				
		Total RoM			
		Decreased	Barrett et al., 1999, Dunk and Callaghan, 2010, Kim et al., 2013, Lariviere et al., 2000, Dankaerts et al., 2009 (in active extension pattern LBP), McGregor et al., 1997 (left and right), Mellin, 1990 (male only), Vaisy et al., 2015, Vismara et al., 2010, Wong and Lee, 2004		
		Increased	Jandre Reis and Macedo, 2015, Kim et al., 2014, Kim and Yoo, 2015 (in flexion pattern LBP), Dankaerts et al., 2009 (in flexion pattern LBP)		
				Upper lumbar segment	
				Decreased	Jayaraman et al., 1994
				Lower lumbar segment	
				Decreased	Jayaraman et al., 1994

	Transverse plane (axial rotation)				
		Total RoM			
		Decreased	Boline et al., 1992, Shum et al., 2005b, Wong and Lee, 2004		
		Increased	Sung et al., 2012		
			Left rotation RoM		
			Decreased	Boline et al., 1992, Sung et al., 2012, Shum et al., 2007a (In LBP and a positive SLR sign on the right), Vaisy et al., 2015	
			RIGHT ROTATION RoM		
			Decreased	McGregor et al., 1997, Vaisy et al., 2015	
			Increased	Shum et al., 2007a	
		Axial rotation at mid-stance			
		Increased	Crosbie et al., 2013a		
		Axial rotation mean			
				Upper lumbar segment	
				Decreased	Gombatto et al., 2015
				Lower lumbar segment	
				Decreased	Gombatto et al., 2015

	Lumbar/hip ratio				
		Total			
		Decreased	Freddolini et al., 2014a, Henchoz et al., 2013, Shum et al., 2005a, Shum et al., 2005b, Wong and Lee, 2004		
		Increased	Kim et al., 2013 (for LBP with lumbar flexion rotation syndrome), McClure et al., 1997		
		Different (values not shown)	Lariviere et al., 2000		
		At 30 to 60° of forward bending			
		*Decreased*	Esola et al., 1996		

RoM:Range of Motion.

**Table 4:** Significant shoulder, pelvis, hip and knee angles reported in the selected studies (corresponding reference number given).

Shoulder joint				
	Coronal plane (lateral flexion)			
		FRONTAL RoM		
		Decreased	Vismara et al., 2010	
	Transverse plane (axial rotation)			
		External rotation RoM		
		Decreased	Mellin, 1990 (male)	

Pelvis segment				
	Sagittal plane			
		Tilt RoM		
		Decreased	Dankaerts et al., 2009 (in flexion pattern LBP), Jandre Reis and Macedo, 2015, Song et al., 2012, Vaisy et al., 2015	
		Increased	Kim et al., 2014	
	Coronal plane (lateral flexion)			
		Obliquity RoM		
		*Decreased*	Crosbie et al., 2013a	
	Transverse plane (axial rotation)			
		Rotation RoM		
		Decreased	Muller et al., 2015, Park et al., 2012	
		Increased	Seay et al., 2011	

Hip joint				
	Sagittal plane			
		Total RoM		
		Decreased	Sung, 2013, Vogt et al., 2003	
			Extension RoM	
			Decreased	Mellin, 1990 (female only), Wong and Lee, 2004 (LBP with restricted straight leg raise)
			Flexion RoM	
			Decreased	Kim et al., 2014, Mellin, 1990 (male only), Shum et al., 2005b (Group 3 subject with LBP and positive straight leg raise sign), Shum et al., 2007a, Wong and Lee, 2004
			Increased	Crosbie et al., 2013a, Fenety and Kumar, 1992
		Mean		
		Decreased	Kim et al., 2013 (lumbar flexion with rotation subgroup)	
	Coronal plane (lateral flexion)			
		Adduction ROM		
		Increased	Shum et al., 2007a	
		Abduction ROM		
		Increased	Shum et al., 2007a (In LBP and a positive SLR sign on the right)	
	Transverse plane (axial rotation)			
		Internal rotation RoM		
		Decreased	Shum et al., 2007a	
		External rotation RoM		
		Decreased	Mellin, 1990 (female only), Shum et al., 2007a	

Knee joint				
	Sagittal plane			
		Extension RoM		
		Increased	Muller et al., 2015 (at heel strike)	
		Flexion RoM		
		Decreased	Sanchez-Zuriaga et al., 2011	

RoM:Range of Motion.

**Table 5:** Significant trunk and thoracic spine angles reported in the selected studies (corresponding reference number given).

Thoracic segment					
	Sagittal plane				
		Total RoM			
		Decreased	Vismara et al., 2010 (also at the instant of max flexion)		
		Increased	Lariviere et al., 2000		
			Extension RoM		
			Decreased	Mellin, 1990(female only)	
			Flexion RoM		
			Decreased	Sanchez-Zuriaga et al., 2011	
				Lower thoracic segment	
				Flexion RoM	
				Decreased	Crosbie et al., 2013b
				Upper thoracic segment	
				Extension RoM	
				Decreased	Crosbie et al., 2013b
	Coronal plane (Lateral Flexion)				
		Total RoM			
		Decreased	Vismara et al., 2010		
				Lower thoracic segment	
				Decreased	Al-Eisa et al., 2006a, Jayaraman et al., 1994
				Upper thoracic segment	
				Decreased	Jayaraman et al., 1994
		Lateral flexion at mid-stance			
				Lower thoracic segment	
				Decreased	Crosbie et al., 2013a
	Transverse plane (axial rotation)				
		Total RoM			
				Lower thoracic segment	
				Decreased	Al-Eisa et al., 2006a, Al-Eisa et al., 2006b
				Upper thoracic segment	
				Decreased	Sung et al., 2012
		Axial rotation at mid stance			
				Lower thoracic segment	
				Decreased	Crosbie et al., 2013a

Trunk segment					
	Sagittal plane				
		Total RoM			
		Decreased	van Wingerden et al., 2008		
	Transverse plane (axial rotation)				
		TOTAL RoM			
		Decreased	Muller et al., 2015		

RoM:Range of Motion.

**Table 6:** Significant angular speed and acceleration at different body segments reported in the selected studies (corresponding reference number given).

Trunk segment				
	Sagittal angular acceleration average & peak			
		Decreased	Aluko et al., 2011	
			Peak angular acceleration in extension	
			Decreased	Sanchez-Zuriaga et al., 2011
			Peak angular acceleration in flexion	
			Decreased	Sanchez-Zuriaga et al., 2011
	Coronal angular acceleration average & peak			
		Decreased	Aluko et al., 2011	
	Axial rotation angular acceleration average & peak			
		Decreased	Cyteval et al., 1996	
	Angular velocity in flexion average & peak			
		Decreased	Sanchez-Zuriaga et al., 2011	
	Angular velocity in extension average & peak			
		Decreased	Sanchez-Zuriaga et al., 2011	

Upper thoracic segment				
	Coronal angular velocity peak			
		Decreased	Jayaraman et al., 1994	

Lower thoracic segment				
	Coronal angular velocity peak			
		Decreased	Jayaraman et al., 1994	
	Peak angular velocity in flexion			
		Decreased	Crosbie et al., 2013b	

Lumbar segment				
	Flexion angular velocity average			
		Decreased	Lee et al., 2011b, Shum et al., 2005a, Shum et al., 2005b, Shum et al., 2007a, McGregor et al., 1997, Vaisy et al., 2015	
	Extension angular velocity average			
		Decreased	Lee et al., 2011b, Shum et al., 2005a, McGregor et al., 1997, Vaisy et al., 2015	
		Increased	McClure et al., 1997 (only during the first interval of extension)	
	Axial rotation angular velocity average			
		Decreased	McGregor et al., 1997, Shum et al., 2007a	
	Coronal angular velocity average			
		Decreased	McGregor et al., 1997	

Upper lumbar segment				
	Coronal angular velocity average			
		Decreased	Jayaraman et al., 1994	

Hip joint				
	Flexion angular velocity average & peak			
		Decreased	Shum et al., 2005a, Shum et al., 2005b, Shum et al., 2007a	
		Increased	Crosbie et al., 2013b	
	Extension angular velocity average			
		Decreased	Shum et al., 2005a	
	Internal rotation angular velocity average			
		Decreased	Shum et al., 2007a	
	External rotation angular velocity average			
		*Decreased*	Shum et al., 2007a	

Pelvis segment				
	Tilt angular velocity average			
		Decreased	Vaisy et al., 2015	
Knee joint				
	Angular velocity in flexion average & peak			
		Decreased	Sanchez-Zuriaga et al., 2011	
	Angular velocity in extension average & peak			
		Decreased	Sanchez-Zuriaga et al., 2011	

## 4 Discussion

This review evaluated studies that reported kinematic and kinetic measures in people with and without non-specific LBP while performing different functional tasks. To our knowledge this is the first review of this type. Two reviews were identified that compared kinematic and kinetic outcomes between control and LBP groups but looked only at balance (Mazaheri et al., 2013) by means of postural sway changes, or at lumbo-pelvic kinematics (Laird et al., 2014).

The search yielded a large number of articles of which 62 were deemed eligible and included in the quality assessment and data extraction. Sources of bias that may expose studies to errors were observed through the qualitative assessment. None of the studies reported the source populations from which participants were recruited and only few indicated if the participants from the two groups were recruited from the same setting, with most studies using samples of convenience. These, together with the small sample sizes described, may limit the validity of the populations selected. Concerns also arise from lack of assessors' blinding, omission of sample size calculations and poor experimental protocol consistency (use of standardized instruction, assessor expertise, same assessors conducting test sessions), which may affect the accuracy of the outcomes and their interpretation.

Based on the studies included within this review, no conclusive statements can be drawn regarding what kinematic and/or kinetic measures should be used to assess LBP. However, few considerations are outlined in the following that could help the design of future studies involving a LBP population. The lack of a clear consensus among the studies may relate to the heterogeneous nature of the studies reporting different body segments modelled, testing procedures adopted, and outcomes evaluated. Inconsistent findings reported may also be explained by the diverse methodologies and tasks performed. While most of the studies assessed participants during RoM maneuvers, several studies reported outcomes during activities of daily living, most commonly walking. Demanding functional activities, such as sit-to-stand, stairs negotiation and lifting, that may present LBP participants with a challenge or be provocative for them, may highlight differences when compared to controls and the review supported this: greater differences were found in studies assessing tasks other than walking (Crosbie et al., 2013a; Gombatto et al., 2015; Lamoth et al., 2006a; Lamoth et al., 2006b; Lee et al., 2011a; Sanchez-Zuriaga et al., 2011; Seay et al., 2011; Shum et al., 2005a; Shum et al., 2005b; Shum et al., 2007a; Shum et al., 2007b; Sung, 2013; Taylor et al., 2003; Taylor et al., 2004; van den Hoorn et al., 2012). This suggests that functional tasks should be considered in the assessment of LBP patients.

RoM was the primary outcome measure reported in the selected studies independent of the tasks and body segments/joints analysed. Despite RoM being a simple metric that could be easily estimated within a clinical setting, it does not convey the contribution over time of the related segments/joints to the movement performed, compensatory actions nor the movement variability, thus limiting our understanding of movement strategies (Al-Eisa et al., 2006a; Al-Eisa et al., 2006b; Needham et al., 2014; Needham et al., 2016). Similarly, this applies to average values over the entire task. Only 16 studies reported the time varying waveforms of the outcomes, thus providing a more comprehensive description of the movement evaluated (Al-Eisa et al., 2006a; Al-Eisa et al., 2006b; Crosbie et al., 2013b; Jayaraman et al., 1994; Kim et al., 2013; Lamoth et al., 2002; Lamoth et al., 2006a; Lee et al., 2011a; Lee et al., 2011b; McGregor et al., 1997; Muller et al., 2015; Park et al., 2012; Seay et al., 2011; Vaisy et al., 2015; van den Hoorn et al., 2012; Vogt et al., 2001). Significant differences in RoM values, between control and LBP groups, were reported for different segments/joints among the studies included and often disagreements were observed. This, together with the above mentioned limitations pushes towards the use of other parameters, with suggestions from reviewed papers to consider asymmetry of motion, angular velocity and acceleration (Al-Eisa et al., 2006a; Al-Eisa et al., 2006b; Burnett et al., 2004; Crosbie et al., 2013b; Dankaerts et al., 2009; Larivière et al., 2002; Lee et al., 2011b; Vaisy et al., 2015) as also supported in another recent review (Laird et al., 2014).

Likewise poor consistency was found in the body segments/joints analysed as well as in which segment/joint significant differences were found among studies. Technology has grown over the past few years facilitating the recording of motion data from several body segments. This allows advanced movement analyses to be performed, however, the current literature has not reflected this opportunity. Although most studies used complex 3-D motion capture systems, only one investigated the whole-body movement despite current recommendations (McGregor and Hukins, 2009; Song et al., 2012). The main focus of LBP assessment still appears to be on the lumbar spine that is sometimes assessed in conjunction with the hip joint and/or thoracic segments. The studies that found differences in segments other than the lumbar indicate that limiting the analysis to the lumbar region is a shortfall. From this review, it is therefore advised, to not limit the analysis to the lumbar region. Moreover, partitioning of the lumbar region into two independent segments is also advocated (Burnett et al., 2004; Dankaerts et al., 2009; Gombatto et al., 2015; Jayaraman et al., 1994; Mitchell et al., 2008).

Some papers accounted for the heterogeneous nature of LBP by subgrouping LBP participants based on specific motor control impairments and found accentuated differences from controls (Dankaerts et al., 2009; Gombatto et al., 2015; Mitchell et al., 2008; Shum et al., 2005a; Shum et al., 2005b; Shum et al., 2007a; Shum et al., 2007b). This highlights how different mechanisms of pain exist and the effects they have on function. Subgrouping LBP participants, based on directional pattern of movement impairments, is worth consideration in future studies, and is an important factor to take into account in the interpretation of kinematic/kinetic measures against healthy controls. Analogously grouping participants homogeneously by their age may enhance differences (Cyteval et al., 1996; Intolo et al., 2009; Sung et al., 2012). Another suggestion could be to group patients by their level of fear of movement as this can pose a limitation to functional movement assessment and fear can produce results not indicative of the actual patients' movement potential.

Regarding clinical translation, portable technologies that would allow the same or analogous measurements collected with laboratory-based equipment would need to be explored; only a few studies employed such technologies (Dunk and Callaghan, 2010; Gioftsos and Grieve, 1996; Larivière et al., 2011; Lee et al., 2011a; Lee et al., 2011b; Morlock et al., 2000; Taylor et al., 2003; Taylor et al., 2004; Van Hoof et al., 2012; Vogt et al., 2003). The main advantage in the use of portable technology is that they allow monitoring over time and in everyday environments where pain usually arises. LBP is often linked with the workplace; monitoring in an everyday setting will offer clinicians objective information to enhance their understanding of LBP causation and to prevent the occurrence of new episodes. Wearable technologies are easy to use, less expensive and time-consuming to operate than laboratory based equipment, however, further development in such technologies is required for clinical translation, particularly in relation to monitoring spinal function (Papi et al., 2017). Understanding what to measure also play a key role in adopting wearable technologies into clinical settings and hence it is important to identify a small number of significant kinematic/kinetic measures from current detailed whole-body biomechanics analysis obtained in research laboratories. Another way to clinically translate the detailed biomechanical analysis is to identify simple functional tests based on simple objective metrics that correlate with detailed kinematic/kinetic measures and use them for clinical purposes with the minimum technology resource needed. However, this requires further study; first to identify key measures, as the current literature fails to do so; and secondly to identify functional tests that could be easily implemented in fast pace clinical settings.

The following limitations should be mentioned about the current review: the search was limited to three databases albeit integrated by reference lists and hand searches to identify the most of the relevant papers; only English-written studies were considered due to lack of translation sources posing a language bias to papers selection; and the quality assessment was based on a customised checklist whose validity and reliability was not assessed (although this was constructed on the lines of the most appropriate checklists for this study). Findings reported in this review should be cautiously interpreted due to the biases identified in the included studies, the majority of which were underpowered.

To conclude, the reviewed studies provided insufficient evidence to identify clear-cut measures that could be used to assess LBP. This review, however, could serve as guidance for future studies involving LBP groups. Based on the findings of the current study, it is advised to consider sound sample sizes of homogenous participants, parameters other than only RoM such as angular velocity, acceleration and time varying waveforms, assess participants while performing daily activities, in particular those critical for LBP, use standardized instructions, include in the assessment the whole-body and consider sub-partitioning of the spine segments. Following these suggestions may help to identify objective measures of LBP in future movement analysis studies.

The following are the supplementary data related to this article.

Supplementary File 1 PubMed search strategy. Supplementary File 1

Supplementary File 2 Assessment checklist questions and correspondent decision rules. Supplementary File 2

Supplementary File 3 Data extraction of included study. Supplementary File 3
