# Supplementary material for: Is there evidence to use kinematic/kinetic measures clinically in low back pain patients? A systematic review
Source: Clin Biomech (Bristol). 2018 Jun;55:53–64. doi: 10.1016/j.clinbiomech.2018.04.006 (PMC6161016; doi:10.1016/j.clinbiomech.2018.04.006)
Supplement: Supplementary File 1 — PubMed search strategy. [file mmc1.docx]

The following search strategy was used in the PubMed database:

((((((((((low back pain[MeSH Terms]) OR low back ache) OR lower back pain)) OR non specific back pain)) OR lumbago))

AND (((((lumbosacral region[MeSH Terms]) OR lumbosacral region*) OR lumbar region*) OR lumbar) OR lumbar spine))

AND (((((((((((((((((((((((((((((((((((((((((((((((((((prognostic factor*) OR predictive) OR risk factors[MeSH Terms]) OR early identification) OR diagnosis) OR functional assessment*) OR disability evaluation[MeSH Terms]) OR disability evaluation*) OR physical examination[MeSH Terms]) OR physical examination*) OR exercise therapy) OR patient satisfaction) OR patient outcome assessment[MeSH Terms]) OR patient outcome* AND assessment*) OR physical fitness) OR exercise*) OR exercise test*) OR gait[MeSH Terms]) OR walking) OR lifting) OR sit to stand) OR twisting) OR binding) OR weight lifting[MeSH Terms]) OR weight lifting*) OR repetitive lifting) OR recovery of function[MeSH Terms]) OR function recover*) OR motor activities[MeSH Terms]) OR locomotor activit*) OR differential diagnosis[MeSH Terms]) OR differential diagnos*) OR physical activit*) OR activities of daily living[MeSH Terms]) OR daily living activit*) OR outcome assessment health care[MeSH Terms]) OR outcome* AND assessment*) OR outcome* AND measure*) OR function*) OR movement analysis) OR motion analysis) OR accelerometry) OR objective tool*) OR data interpretation, statistical[MeSH Terms]) OR statistical data analys*) OR statistical data interpretation*) OR data analys*) OR objective measure*) OR objective assess*) OR shuttle walking test) OR sorensen test))

AND (((((((((((((((((((((((((((((((((((((((treatment outcome[MeSH Terms]) OR treatment effectiveness) OR clinical efficacy) OR treatment efficacy) OR rehabilitation outcome*) OR patient relevant outcome*) OR posture) OR body posture) OR lumbar posture) OR pain) OR spinal function) OR spinal stiffness) OR activity level) OR movement) OR spine motion) OR lumbar motion) OR biomechanics) OR musculoskeletal) OR balance) OR range of motion, articular[MeSH Terms]) OR joint range of motion) OR range of motion) OR joint flexibility) OR outcomes) OR functional capacity) OR kinematics) OR joint angle*) OR lumbar angle*) OR thoracic angle*) OR spine kinematics) OR spinal kinematics) OR thoracic kinematics) OR joint force*) OR muscle fatigue[MeSH Terms]) OR muscle strength) OR kinetics) OR joint moment*) OR acceleration) OR joint coordination)
