# Supplementary material for: Is there evidence to use kinematic/kinetic measures clinically in low back pain patients? A systematic review
Source: Clin Biomech (Bristol). 2018 Jun;55:53–64. doi: 10.1016/j.clinbiomech.2018.04.006 (PMC6161016; doi:10.1016/j.clinbiomech.2018.04.006)
Supplement: Supplementary File 2 — Assessment checklist questions and correspondent decision rules. [file mmc2.docx]

# Quality Assessment checklist

|  | **Criteria** | **Decision rule** |
| --- | --- | --- |
| **Study population bias** | | |
|  | Was the study population adequately described? | Age (mean±SD, or range), gender (male vs female subjects), and anthropometric information (at least one of the following: body mass, height, BMI). All three variables must be included to score yes. |
|  | Were both groups drawn from the same population? | People were from the same setting, eg people with and without back pain from a single setting such as a university, OR were they matched age/gender/BMI or weight case controls. If so score yes, if no or no data, score no. |
|  | Were both groups comparable for age, sex, BMI/weight? | Was a comparison made between groups on these parameters? Yes if comparison made AND groups were comparable. No if not comparable or no comparison made. |
|  | Were the subjects asked to participate in the study representative of the entire population from which they were recruited? | The study must identify the source population for patients and describe how the patients were selected. Patients would be representative if they comprised the entire source population, an unselected sample of consecutive patients, or a random sample. Random sampling is only feasible where a list of all members of the relevant population exists. Where a study does not report the proportion of the source population from which the patients are derived, the question should be answered no. |
|  | Was pain intensity and/or activity limitation described for LBP group? | Score yes if measured using a validated scale, such as a Visual Analogue Scale or Numeric Rating Scale for pain or the Oswestry Disability Index or Roland Morris Disability Questionnaire or similar. |
|  | Was an attempt made to define back pain characteristics? | (i) Stage (acute/subacute/chronic) (ii) +/- leg pain? (iii) Score yes if information provided |
|  | Were the eligibility criteria specified? | Yes if inclusion and exclusion criteria were clearly stated |
| **Measurement and outcome bias** | | |
|  | Did the method description enable accurate replication of the measurement procedures? | Description enables accurate replication of the measurement procedures (score yes). |
|  | Was the measurement equipment adequately described? | Instrument used to measure described (score yes). |
|  | Was a system for standardizing movement instructions reported? | A system for standardizing movement instructions is reported (score yes). |
|  | Were assessors trained in standardized measurement procedure? | Yes if report of training, or no, if no mention of training process. |
|  | Did the same assessors test those with and without back pain? | If yes then score yes. If no detail score no. |
|  | Were assessors blinded as to which group subjects were in? | If blinding attempted, was it evaluated and found to be successful (e.g. attempting to guess group assignment resulted in answers that could occur by chance alone). |
|  | Was assessment procedure applied to those with and without back pain the same? | If there was any difference to procedure or measurement then score no. |
|  | Were the main outcomes to be measured and the related calculations (if applicable) clearly described? | Score yes if details of outcomes and how they were obtained from the collected data are reported. |
|  | Were the main outcome measures used accurate (valid and reliable)? | For studies where the outcome measures are clearly described, the question should be answered yes. For studies which refer to other work or that demonstrates the outcome measures are accurate, the question should be answered as yes. |
| **Data presentation bias** | | |
|  | Are the main findings of the study clearly described? | Simple outcome data should be reported for all major findings so that the reader can check the major analyses and conclusions. (This question does not cover statistical tests which are considered below). |
|  | Were the statistical tests appropriate? | Yes or no. |
|  | The results of between-group statistical comparisons were reported for at least one key outcome | Yes or no. |
|  | Have actual probability values been reported (e.g. 0.035 rather than <0.05) for the main outcomes except where the probability value is less than 0.001? | Yes or no. |
|  | Point estimates and measures of variability were provided for at least one key outcome for those with and without back pain | Yes or no. |
|  | Did the study have sufficient power to detect a clinically important effect where the probability value for a difference being due to chance is less than 5%? | Sample sizes have been calculated to detect a difference of x% and y% (score yes). |
|  | Was the reliability and/or validity of the outcomes commented upon? | Yes or no. |
